# Software Defined Networks in Wireless Sensor Architectures

**DOI:** 10.3390/e20040225

**Published:** 2018-03-26

**Authors:** Jesús Antonio Puente Fernández, Luis Javier García Villalba, Tai-Hoon Kim

**Affiliations:** 1 Group of Analysis, Security and Systems (GASS), Department of Software Engineering and Artificial Intelligence (DISIA), Faculty of Computer Science and Engineering, Office 431, Universidad Complutense de Madrid (UCM), Calle Profesor José García Santesmases, 9, Ciudad Universitaria, 28040 Madrid, Spain; 2 Department of Convergence Security, Sungshin Women’s University, 249-1 Dongseon-Dong 3-ga, Seoul 136-742, Korea

**Keywords:** controller, data plane, management, resource allocation, software defined networks, wireless sensor networks

## Abstract

Nowadays, different protocols coexist in Internet that provides services to users. Unfortunately, control decisions and distributed management make it hard to control networks. These problems result in an inefficient and unpredictable network behaviour. Software Defined Networks (SDN) is a new concept of network architecture. It intends to be more flexible and to simplify the management in networks with respect to traditional architectures. Each of these aspects are possible because of the separation of control plane (controller) and data plane (switches) in network devices. OpenFlow is the most common protocol for SDN networks that provides the communication between control and data planes. Moreover, the advantage of decoupling control and data planes enables a quick evolution of protocols and also its deployment without replacing data plane switches. In this survey, we review the SDN technology and the OpenFlow protocol and their related works. Specifically, we describe some technologies as Wireless Sensor Networks and Wireless Cellular Networks and how SDN can be included within them in order to solve their challenges. We classify different solutions for each technology attending to the problem that is being fixed.

## 1. Introduction

Communication technologies have experienced a large evolution since the 1980s to become what are currently Software Defined Networks (SDNs). Nevertheless, there are some concepts that shape the base of this technology. On one hand, these advances are: central control, active networks and network virtualization. On the other hand, the great progress that has been achieved is in the control-data plane separation. Centralized control goes back to the beginning of the 1980s, and it focused on the transport of information over the same channel. This technology offered several advantages in terms of simplicity; however, it was quite fragile, insecure and vulnerable.

Active networks [1,2] appeared during the 1990s. These kinds of networks allow for performing custom assignments in the data packets that travel around the switches. The most popular example of active networks is middleboxes (e.g., firewalls), which are mainly in charge of packet forwarding, proxy functions and application services. Finally, network virtualization [3,4] plays an important role in the management of setting up these types of networks. Networks virtualization has similarities to computers’ virtualization, where different devices can use and share hardware resources to different Operating Systems (OS) in a host. The aim of network virtualization is to isolate multiple logical networks, each of them with addressing and forwarding mechanisms, sharing the same physical infrastructure. Clear instances of network virtualization are Virtual Local Area Networks (VLANs).

SDN addresses the shortcoming of the previous technologies by separating the data plane and the control plane. Particularly, switching elements in SDN involve data plane functionality (forwarding) since they are controlled and configured by a centralized controller. Then, controllers serve as central points to gather network view and network information to configure instructions and decisions on how the network resources should behave.

Currently, SDN is being investigated in the field of wired provider networks, data centers and research networks among others. Deployment of large SDN testbeds as the one performed by the Ofelia consortium [5] and the Open Networking Foundation (ONF) [6] have increased the interest in this technology. Nevertheless, such deployments are applying to other type of networks in order to solve existing challenges. This paper provides a survey and categorization of some existing wireless networks and the application of SDN to them. For each technology, we overview, classify, contrast and summarize possible SDN solutions to solve challenges as resource allocation and efficient management.

New emerging technologies as Fog computing [7,8], alternatively known as fog networking or “fogging”, is a term originally introduced by Cisco (San Francisco, CA, United States of America) that follows the SDN principles. Similar to SDN, Fog computing consists of a control and a data plane. The data plane enables computing services located in the edge of the network. Then, Fog Computing provides compute, storage and network services and networking services between end devices and traditional Cloud Computing Data Centers, making reference to the growing difficulties in accessing information. The main feature of Fog Computing is the accentuation on the end-users’ proximity and client objectives, dense geographical distribution and local resource pooling to achieve better Quality of Service (QoS). Then, Fog Computing uses multiple cloud platforms introducing a logical architecture from the point of view of processing, considering a federation of cloud platforms with the objective to specify fog-to-fog and fog-to-cloud interfaces. Moreover, Fog networking supports the Internet of Things (IoT) concept overall in the connection between the world population with their devices, taking advantage of its main feature previously described. Examples of this connection includes smartphones, wearable devices and the vehicles’ connectivity among others. The SDN concept can be applied to Fog Computing, at a more abstract level, allowing centralized control over computation, storage and network resources. Gupta et al. [9] propose extending the Fog Computing with the concept of SDN in the application layer, resulting in “Software Defined Fog” (SDFog). This approach is able to execute functions in the data plane dealing with the resources on fog nodes.

Other emerging technology that enables IT and cloud Computing capabilities at the Radio Access Network (RAN) edge in a close proximity to the end users is the Multi-Access Edge Computing (MEC) [10]. IT provides an ultra-low latency environment and realtime access to radio and network analytics. Then, the use of MEC offers contextual information and specific content, proximity and location awareness, providing a customized mobile broadband experience. Moreover, it collects information from customers in terms of content, location and interests to introduce new services or simply use such information for commercial reasons.

MEC is a key enabler for supporting Machine-to-Machine (M2M) and IoT services due to the ubiquitous coverage of cellular networks. For example, providing network scalability and reducing IoT data and signalling, ensuring a fast response to user requests or enabling new services. From the business point of view, MEC enables a secure cloud platform architecture and Application Programming Interfaces (APIs) to allow third players to share and use it dynamically, easily installing and modifying new services. As mentioned before, MEC relies on cloud computing and virtualization technologies including Virtual Machines (VMs) and containers. Nevertheless, a complete cellular system that offers MEC services is supported by Network Function Virtualization (NFV) and SDN, and network slicing attributes that allow flexibility and multi-tenancy support. Then, SDN can complement the use of MEC offering programmability capabilities to authorized tenants at the same time, which allows a flexible and efficient service management.

We start with the Wireless Sensor Networks (WSN) [11], which is a technology that has become very important in recent years because of the quick and cheap development of smart sensors. This fact is possible with the innovation in the production chains of Micro-Electro-Mechanical Systems (MEMS). Smart sensors, which are developed following this technology, are smaller, cheaper and limited in terms of processing and computing resources compared to traditional sensors. The main property of these sensors is the low power consumption among others, as information collection from the environment where they are deployed. A smart sensor is based on a processor, a memory, a power supply, a radio and an actuator, which is an electro-mechanical device that is responsible for controlling different components in a system. A WSN topology is comprised from few to thousand sensor nodes that run together in order to gather data from an area or environment. Two types of WSN are defined in [12]: the structured and unstructured. The difference between them is the quantity of sensors that the topology is composed of: the unstructured WSN contains a huge quantity of sensors, while the structured WSN are composed of a small quantity. The main applicability of WSN is categorized in two fields: on one hand, tracking applications and, on the other hand, monitoring applications. The first one includes tracking entities such as animals, humans, vehicles and other types of objects. Monitoring applications include monitoring environmental scenarios, both indoors and outdoors.

Next, Software-Defined Sensor Networks (SDSNs) [13] are the result of taking the leading characteristics of WSN and SDN technologies. SDSNs are based on smart nodes as WSN but including the capability of being programmed on demand (as SDN) by loading, in each case, the application that the SDSN will be used for. Therefore, SDSNs are versatile in terms of changing their purposes into several applications [14]. In addition, they are flexible since they are managed in a centralized way. Then, SDSNs are easily managed due to the simple way to be programmed due to the availability of different open APIs. On the contrary, SDSNs must offer duty cycles since the sensor energy consumption when the sensors are not taking measurements is an important problem to be solved in this technology.

Nowadays, smart phones and tablets have increased the use of cellular networks due to the big demand of such devices. Moreover, despite the huge innovation in mobile applications, the infrastructure of cellular networks is quite fragile. Then, supporting several subscribers, user mobility and real-time adaptation offer some scalability challenges. Finally, we review Wireless Cellular Networks (WCN) [15] providing their architecture and how SDN addresses the problem of this technology.

WCN provides the integration of multiple technologies as WiFi [16] and WiMAX [17] within the same network and also supports mobility between them. The transition process from one technology to another is known as handover. Handovers are classified depending on the technology and the domain: if the handover is performed between two-different network technologies, the handover is categorized as inter-technology, otherwise (both network technologies are the same), it is called intra-technology. Several SDN solutions have been proposed in WCN to improve some challenges as resource allocation, interference mitigation and handover between networks. Further challenges are congestion control and load balancing. We gather SDN designs that use centralized control, virtualization, resource allocation, tasks distribution and layered controllers.

The rest of the paper is outlined as follows. Section 2 provides background on SDN and OpenFlow. This section also reviews related works in SDN and the main Network Operating System that is being used in SDN model. Section 3 describes the Wireless Sensor Networks and their main characteristics. Moreover, the applicability of this type of network is proposed giving a wide view of what they are able to perform. Section 4 presents the union of Wireless Sensor Networks and Software Defined Networks resulting in Software Defined Sensor Networks. Similarly, Section 5 describes Wireless Cellular Networks architectures and the use of SDN models in these networks. Finally, Section 6 concludes with a discussion of the main themes explained in this work and its conclusions.

## 2. Background

In this section, we provide a general overview the general architecture of Software Defined Networks and the protocol that manages them, the OpenFlow protocol. In addition, we present some of the main Network Operating Systems that are currently available to control data flows through the network.

### 2.1. *Software Defined Networks*

Traditional Internet Protocol (IP) networks require trained personnel to manage both the network configuration and the network devices. Moreover, distinct vendor network devices and their maintenance costs propose a huge challenge to be tackled by researchers. At the same time, SDN architecture provides network management in a centralized point called the Controller.

SDN is a new network architecture model that gathers the advances of statements previously mentioned. It separates control planes and data planes in network devices to enable a programmable behaviour removing the rigidity of static protocols. In terms of data planes, SDN differs from traditional IP networks since the packet forwarding is based on flows instead of packets. In addition, it proposes a centralized control of the network by a high-level software application that allows quick network management and quick innovation. Therefore, a network administrator can have a centralized and programmable control of the traffic behaviour within the network without requiring an individual configuration of hardware devices. SDN is used in different scopes as virtualization, home networking, security, data centers, IoT, among others [18]. In this context, OpenFlow [19,20] is the protocol that is the most used in SDN networks. The Open Networking Foundation ONF [6] is the organization responsible for the OpenFlow specification and its maintenance. In this paper, we also describe an introduction of the OpenFlow protocol in next section.

Network virtualization allows the coexistence of different virtual networks within the same underlying network. In other words, a virtual network consists of a set of virtual switches and virtual links [21] that will be used to provide end-to-end network services. Therefore, they are sharing and managing the same underlying resources, but, at the same time, they are isolated from each other. In this way, works like FlowVisor [22] and CoVisor [23] present an extra layer that is located between the data plane and the controller. The difference between both approaches is that FlowVisor provides the existence of a dedicated controller for a set of virtual networks while CoVisor does not restrict such existence to a single controller.

Similarly to network virtualization, the concept of NFV [24,25] separates network services from devices to get a better performance in the network management. Such set of services includes firewalls, monitoring tools, load balancers among others.

**Figure 1:**
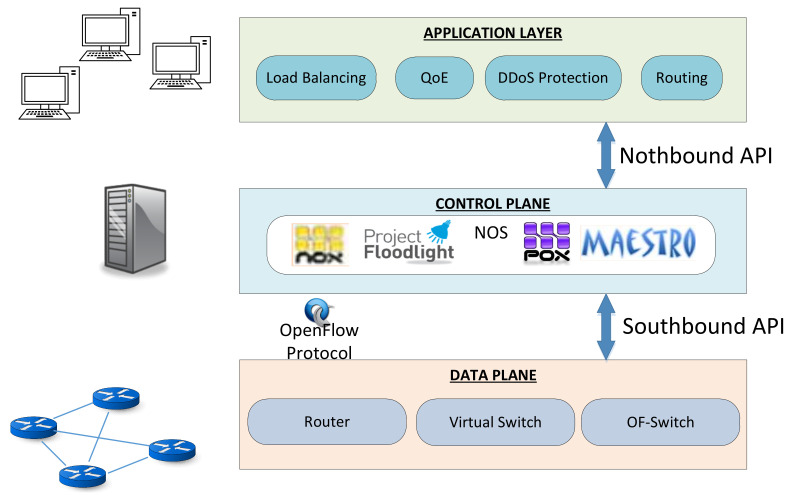
SDN layers.

Some authors [26,27,28] have classified the abstractions of network resources like southbound and northbound interfaces (Figure 1). Southbound interfaces have the ability to abstract the functionality of the programmable switches and connect them to the software that is running in the controller. The most representative example of southbound interfaces is OpenFlow.

Over these types of interfaces a Network Operating System (NOS) is running, which describes all software tools that allow creating applications and controlling the network behaviour. Examples of NOS are NOX/POX [29], Maestro [30], Beacon [31], Floodlight [32], Open Network Operating System (ONOS) [33] and OpenDayLight [34]. Table 1 gathers the main NOS currently used and the languages that are specified. Northbound interfaces allow the creation of applications and high-level network policies. In addition, they are responsible for sending these policies to the NOS. Examples of northbound interfaces are Frenetic [35,36], Procera [37], Netcore [38] and McNettle [39].

SDN presents some challenges in terms of performance, flexibility, scalability and security. In this context, the performance is defined as the processing rate of the nodes that the network is composed of. Moreover, the term flexibility is the feature to fit systems to back up against unexpected features.

Nowadays, several processors’ architectures provide the best solution for the application that is going to be used for. Then, the challenge is to obtain performance and flexibility as much as possible. In order to get the most flexibility, it usually uses General Purpose Processors (GPP), such as the Intel Xeon [40], in implementations, but it also provides the disadvantage that the general purpose architecture is limited in terms of performance. Another choice is to use Network Flow Processors (NFP) [41] that are specialized hardware for processing data in form of packets. This solution furnishes very good performance, but, in contrast, the flexibility is reduced since it requires defining a treatment function for packets. As a result, a hybrid solution is the best option if the applications need both features and, therefore, is the only approach that satisfies SDN.

Continuing from the last statement, the next challenge is how a hybrid architecture can provide scalability at the same time that the network grows up. The scalability can be divided into points of view: controller scalability and switch scalability. The main issue is within the network controller, which experiments with latency belonging to the exchanging of network information between itself and the switches that compose the network. The other is the way of communication between different controllers of distinct networks. Different approaches have been proposed to solve these problems. To solve the latency problem, a possible solution is to deploy an infrastructure with a distributed or peer-to-peer controller that shares the data load of itself. At the same time that this solution solves the issue of the latency, it does not determine the way of communication between controllers. A good solution to manage the scalability is Hyperflow [42], which consists of a controller that uses NOX [29] as NOS, which distributes events about the state of the network.

The final challenge and probably the most important is the security in SDN against attacks. SDN security [43] is focused on which component is the target of the attack. From the controller point of view, some issues exist regarding unauthorized access and impersonation. An attacker who masquerades as the controller could run malware or malicious code and manipulate the entire network as he wants. This trouble is solved using a Transport Layer Security (TLS) with authentication on both sides (controller and switches) in the specification of OpenFlow [20]. Nevertheless, this security feature is optional and an attacker can take advantage of this problem and hijack the communication. From another point of view, network switches are a target of Denial of Service (DoS) due to a possible increase of traffic across the network. Since the incoming packets headers are sent to the controller and also stored in the switch memory, this supposes a traffic jam and an attacker can take advantage of this issue to overload the memory of the switch, and, consequently, it overloads the network.

### 2.2. OpenFlow Architecture

OpenFlow is an open source protocol that provides instructions to program flow tables within switches and routers. Therefore, a network administrator is able to program the traffic across the network and also control its own flows by selecting their routes based on their requirements. In this way, OpenFlow opens up several possibilities in the research community to elaborate new network protocols.

**Figure 2:**
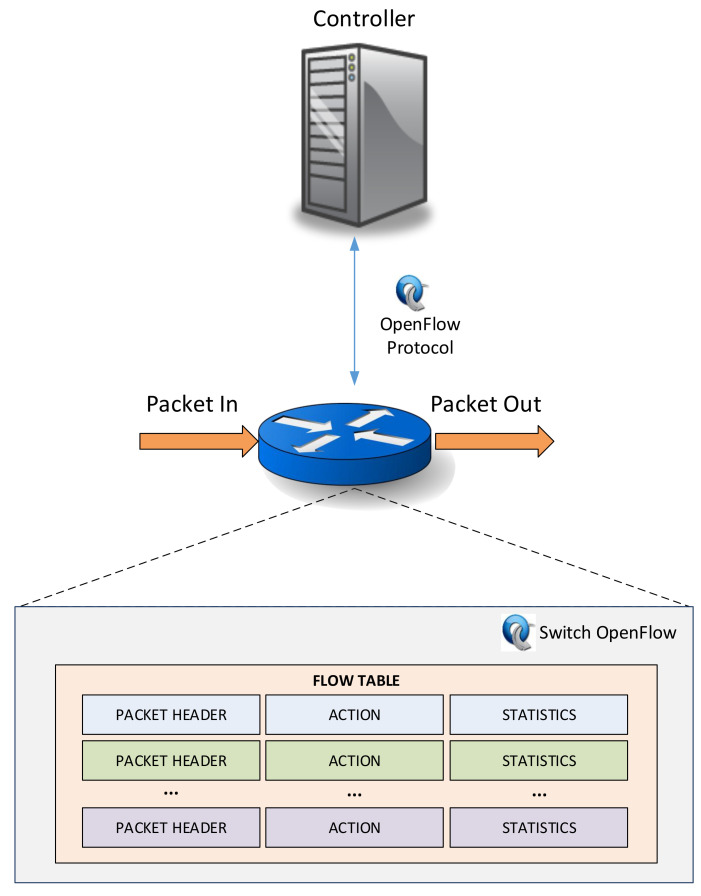
OpenFlow architecture.

The OpenFlow architecture is based on three main entities as is shown in Figure 2: an OpenFlow switch (data plane), an external controller (control plane) and the OpenFlow Protocol [19] that is responsible for the communication between controller and switches through a secure channel.

On a data plane, one or more flow tables and a group table are used for the packet lookup and data forwarding within an OpenFlow switch. Each flow table is based on a group of match fields, counters and instructions. Match fields manage incoming packets that must be processed by the switch or must be sent to the controller through a secure channel in a process called matching. Moreover, in counters, records are saved regarding the number of packets that are matched. Finally, the instructions field determines the actions to be performed when a matching occurs.

OpenFlow switches are divided into two categories attending to the ones (dedicated OpenFlow switches) that do not support processing in Layer 2 and Layer 3 traffic forwarding using traditional distributed control protocols, such as RSTP, LLP, and the ones that are used as general purpose commercial Ethernet switches and routers (OpenFlow-enabled switches). In this way, dedicated OpenFlow switches forward incoming packets to the port that the controller has assigned to them. In contrast, OpenFlow-enabled switches are normal commercial switches that are provided with the OpenFlow architecture adding flow tables, secure channel and the OpenFlow protocol.

The packet treatment is performed in a process called matching. This process is divided into two steps: the first consists of checking the packet header with the match table of a single flow table. The second step depends on the result of the step one. If these two values (packet header and match table) are similar, the corresponding actions of the instructions field are taken. Otherwise, the OpenFlow Protocol specifies the required and optional actions that a single switch can take. Three possible instructions compose the required actions: send the packet by a given port, send the packet to the controller or drop the packet. Even these actions are obligatory, in the case where the incoming packet does not match with a match field, the switch can be configured to send it to the controller or drop it.

### 2.3. Network Function Virtualization

As mentioned before, SDN and NFV are two important newness in network architectures. Although these technologies were developed individually, their combination opens up new network designs. Such combination is possible due to the common goals that they share that are complementary to each other. In this way, integrating both SDN and NFV is the target of many research efforts and industries in order to take advantage of their benefits. Nevertheless, this integration is becoming a big challenge due to the diverse elements implied in their interactions. Then, hypervisor and virtualization mechanisms have been used for supporting multiple virtual SDN networks. An example of this research approach is Flow Visor [3], which is previously explained.

Duan et al. [44] analyse the combination of these architectures providing a framework, which is categorized as SoftwareDefined Network Virtualization (SDNV). Equal to SDN, this framework is separated into data with control/management planes.

It is divided into three layers as follows: Infrastructure Layer, Virtualization Layer and Service Layer. Both the Service Layer and the Infrastructure Layer have been separated in the data and control planes.

The Infrastructure Layer (lowest layer) is composed of the network’s physical resources and compute infrastructures. These sets of physical resources and compute infrastructures can be deployed as several autonomous domains (each domain with its proper controller).

On the other hand, the Virtualization Layer (middle layer) provides abstraction in the connection between the Infrastructure Layer and the Service Layer. In addition, it provides a physical and virtual mapping for network resources.

Finally, the Service Layer provides (upper layer) service-related functionalities. It receives the virtual resources provided by the Virtualization Layer to carry out the following functions: Virtual Service Functions (VSFs), Virtual Network Functions (VNFs) and Virtual Compute Functions (VCFs). Using these functions, the Service Layer selects and orchestrates the appropriate functions to build Virtual Networks (VNs) to satisfy users’ requirements.

## 3. Wireless Sensor Networks

This section reviews the general architecture of Wireless Sensor Networks and their main characteristics. Moreover, we present the applicability of these types of networks in different fields of real life, and, finally, we describe some challenges that are placed as a consequence of this technology.

### 3.1. Wireless Sensor Networks Architecture

**Figure 3:**
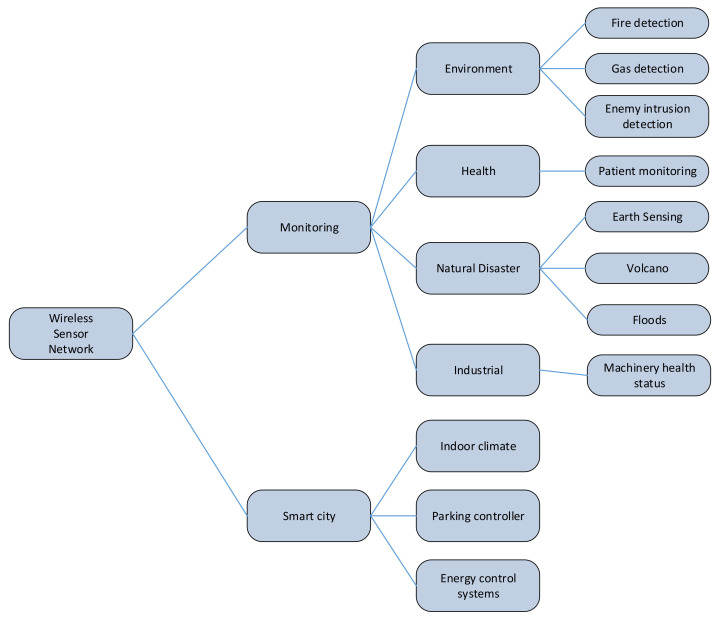
Wireless sensor network architecture.

Wireless Sensor Networks (WSN) [11,45] are typically a set of distributed autonomous sensors that are used to monitor physical or environmental conditions such as temperature, sound, and humidity, among others. WSNs are composed of a few or several devices called nodes, which contain a radio transceiver with an internal antenna, a microcontroller and an energy source. A WSN is also composed of a base station that stores the collected data and an application server as Figure 3 depicts.

A WSN includes several characteristics such as:

- Fault tolerant: nodes have the ability to cope with node failures.
- Heterogeneity of nodes: all nodes do not have to contain the same brand or the same characteristics.
- Energy harvesting: power consumption constraints for nodes using batteries.
- Durability: ability for resilience hard environmental conditions.
- Scalability: big deployments of nodes do not interfere in the performance of the network.

In order to test these types of networks, there are some available simulators to check its behaviour. For example, simulators as OPNET [46], NetSim [47] or NS2 [48] are the most known nowadays.

### 3.2. Wireless Sensor Networks Applicability

**Figure 4:**
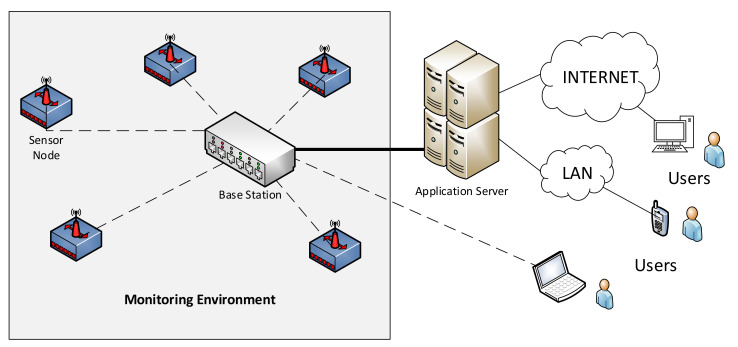
Summary of WSN applicability.

WSNs are highly useful in several fields of research, overall in computer science and telecommunications. Such networks provide a great applicability from the point of view of monitoring to obtain important information in a system or an environment. Figure 4 provides an organized tree of WSN applicability.

The most common application of WSN is area monitoring, which consists of deploying sensors in a place to detect some phenomenon that wants to be monitored. An example of area monitoring is the use of this type of network to detect fire, gas, enemy intrusion among others.

WSN are also used in smart grids and energy control systems in order to measure the quantity of energy that a user or application is using, and, at the same time, they are able to regulate such energy consumption. In this way, WSNs are extremely useful in smart buildings at controlling the indoor climate, controlling parking lots, turning on light bulbs in corridors and so on.

Another great applicability is found in the health care field [49,50]. Medical applications can help patients to detect and to tackle possible diseases with enough time to apply an effective treatment. It is divided into two types: implanted or wearable. Regarding implanted devices, they are composed of the artifacts that are introduced into the human body. Moreover, they monitor human information such as human tension and sugar levels among others. Nowadays, fitness information is gaining importance since the quantity of people practising sports is growing. In this way, other types of sensor networks applicable to health care have appeared, the wearable devices. These devices are used on the surface of the human body surface or close to vital organs, in order to measure data from the body (number of steps in a day, consumed calories, cardiac frequency, etc.).

In addition, other fields in which to apply sensor networks is environmental or earth sensing. There are several examples of these kinds of sensor networks, but the most important are the following: firstly, air pollution monitoring WSNs are deployed in some cities to measure the concentration of dangerous gases for the population. These dangerous gases come from cars and factories among others. The advantage of wireless sensors with respect to the wired sensors is the mobility to test data readings in different places or areas across the city. Secondly, a WSN can be set up in a forest in order to detect if a fire has been initiated. In this case, network nodes have been deployed with sensors to measure the percentage of gases, temperature and humidity. The advantage in this field is the capacity to detect prematurely a fire and how it is expanding across the forest. Thirdly, a WSN is very useful to detect slight movements on the floor. Sensors within network nodes are able to gather data, which is very important to in order to get a pattern or an occurrence of landslides. The advantage in this applicability is the reduction in the reaction time before a soil movement happens. Following this topic, WSN can be effectively used in the prevention of natural disasters like floods [51], hurricanes, volcano eruptions, etc. In this way, Werner et al. [52] propose WSN to study seismic signals in a volcano. The proposed deployment consists of 16 sensor networks with a seismometer and a microphone that gather seismic and acoustic data from the volcano. These sensors send collected data to a gateway node that is also situated near the volcano, and, after that, it transmits the data via free wave radio to an observatory. Fourthly, a direct human involving factor is the water quality. WSNs are established in oceans and rivers as well as underground water reserves. The use of sensor networks provides an accurate water status as input to a system that determines if the water is drinkable.

Another important applicability of WSN is in industrial monitoring. To start with, the preservation of machine health is an important task for which a WSN can help to gather data about the status of such machinery. It offers an important cost savings, and it enables new functionalities due to its ability to be set up in difficult or inaccessible places that a wired network could not offer. Moreover, WSNs are used for collecting environmental information such as the temperature of a fridge and the level of water in overflow tanks in nuclear power plants, among others. Next, monitoring the quality and level of water includes some tasks such as checking the quality of underground or surface water in order to protect the wastage of water. Finally, a WSN can be used to monitor the conditions of civil infrastructure and related geophysical processes in real time.

Multimedia transmissions in WSNs include huge requirements of bandwidth, power consumption, QoS, treatment of gathered data, and routing protocols [53], among others. When a user is required to send a video, it needs an elevated bandwidth to be handled during the transmission. Consequently, this raised requirement is transformed into an increasing energy consumption. Some factors as delay and channel capacity, among others, directly affects the QoS of the network, and, therefore, the video delivery has to achieve a minimum level to ensure good reception of the video.

### 3.3. Wireless Sensor Network Challenges

WSNs present some general challenges [54] in terms of sensor management and data processing unification. One of the problems of WSN is the Network Discovery, since the network topology has to be built in real time and it has to be updated permanently against adding new sensors or sensors’ failures. Another challenge is Network Control because each network node changes dynamically in terms of energy, bandwidth, etc. Routing is also an important aspect of WSN in contrast to IP routing, since the routing paths are established from geoinformation depending on the needs. Moreover, Collaborative Signals and Information Processing require the communication between nodes, and, at the same time, do not lose any signal during the transmission. Tasking and Querying include the methods to query data gathered from the network. Finally, the most important challenge is the Security since the network can be deployed in a hostile environment, and, therefore, it is exposed to physical attacks.

As described before, several surveys have discussed the aspects on WSN. Baronti et al. [55] provide a comprehensive review on most recent developments and challenges that the WSN need to overcome with some solutions to mitigate them. In particular, Ref. [55] also deals with the increasing importance of IEEE 802.15.4 standard [56] in WSNs. Such standard defines the characteristics of the physical and Medium Access Control (MAC) layers for Low-Rate Wireless Personal Area Network providing reliable data transfer, short-range operation and reasonable battery life while maintaining a simple and flexible protocol stack. Four key requirements must be fulfilled for a creditable WSN deployment in industrial environments: energy efficiency, scalability, reliability, and timeliness. The first requirement, energy efficiency, is extremely important since the sensor nodes are typically powered by batteries that some of them cannot be replaced or recharged due to the environmental circumstances. In this case, the 802.15.4 standard includes a power management mechanism, based on duty cycle, to minimize the activity of sensor nodes. The second feature, scalability, is another important factor that has to be considered in a WSN since the number of deployed sensor nodes may be high, especially when large deployments cover big geographical areas. Finally, reliability and timeliness are very critical factors in the industrial field. If a given set of data packets are not correctly delivered to the sink, the correct behaviour of the system can be compromised. In these terms, the IEEE 802.15.4 standard provides a very low reliability in terms of packet delivery ratio when power management is enabled. This behavior is caused by the 802.15.4 MAC protocol, and, therefore, throughout, we will refer to it as the 802.15.4 MAC unreliability problem. Anastasi et al. [57] propose a solution for this issue taking advantage of the flexibility in choosing Carrier Sense Multiple Access / Collision Avoidance (CSMA/CA) parameters that the IEEE 802.15.4 standard offers.

Perrig et al. [58] resume the main security challenges and problems in WSN. They state that a secure system is the one that each one of its components are secure since the ones that do not provide security are potential point of attacks. In addition, they affirm that a secure system has to fulfill some features such as key establishment and trust set up, authentication, privacy, robustness to communication, secure routing and resilience to node capture. Regarding key establishment, the easiest solution is the establishment of a shared key across the network, but, unfortunately, all network traffic can be decrypted if the key is intercepted. The most used choice to provide security between nodes is the public key cryptography. In order to provide protection against attackers who wants to spoof, eavesdrop or inject malicious code, WSNs have to provide authentication using cryptography. Using it, senders and receivers are identified in the network as reliable entities. Nowadays, privacy is the most important factor in communications. The risk of being spied is elevated since there are many reasons to do it, but, at the same time, a set of social rules and laws do not allow it to happen. Robustness to communication can affect the performance of the network through denial of service attacks [59]. This challenge tackles the employment of spread-spectrum communications. Secure routing is also an essential service of WSNs, as the existing routing protocols experience vulnerabilities due to the high sensibility of capturing network nodes [60]. Consequently, the resilience to node capture is also a challenge in order to maintain the security. Solutions to these problems are tamper evident methods included in sensor nodes, collecting data of the environment and crosscheck for consistency among others.

WSNs open a wide range of possibilities in terms of building new applications, creating or improving protocols, introducing new design concepts and developing new algorithms. Ye et al. [61] present a new protocol design that aims to reduce energy consumption, but, at the same time, it provides high scalability preventing collisions. This protocol is composed of three main components among others: a component that is listening and sleeping periodically, a component for preventing collision and eavesdropping, and, finally, a component for sending messages. Periodic listening and a sleeping component tries to decrease the listening time to a periodic value. Then, if the time of listening is cut in half, the energy saved is also reduced by half. To prevent collision and overhearing, it uses the duration field within each packet, which indicates the remaining time for the next transmission, and, then, it avoids packet collision. The proposed message handling divides the message into small fragments that will be sent in burst.

Finally, physical attacks regarding capturing network nodes are also a big problem. In contrast to traditional IP networks that were inaccessible for possible attackers, WSN are placed in environments that are not totally guaranteed.

## 4. Emerging Sensor Networks: Software Defined Sensor Networks

The concept of Software Defined Sensor Networks (SDSN) has appeared due to the need and demand on applications-specific networks and also the decline in the price of sensors. SDSNs consist of sensor nodes (described in Section 3) including the feature of changing dynamically according to software applications (the same as SDN). This section describes the SDSN architecture and the main challenges that this technology presents.

### 4.1. Software Defined Sensor Networks Architecture

Physically, SDN and SDSN architectures are similar in terms of management since the first ones are governed by a controller and the second ones by a sensor control server. The procedure of deploying a new task or application is through reprogramming sensor nodes with the code that a user requires to be run within the network. In addition to the high applicability of WSN (temperature sensing, seismic movement, pollution measurement, etc.), SDSN incorporates a mechanism to change the network configuration with roles. The Sensor Control Server (SCS) is responsible for setting the value of the current role. Then, another entity called a scenario compiler (also placed in the server) generates the role program according to the application that will be used [51,52], and, after that, it will be sent to sensors through wireless communication.

**Figure 5:**
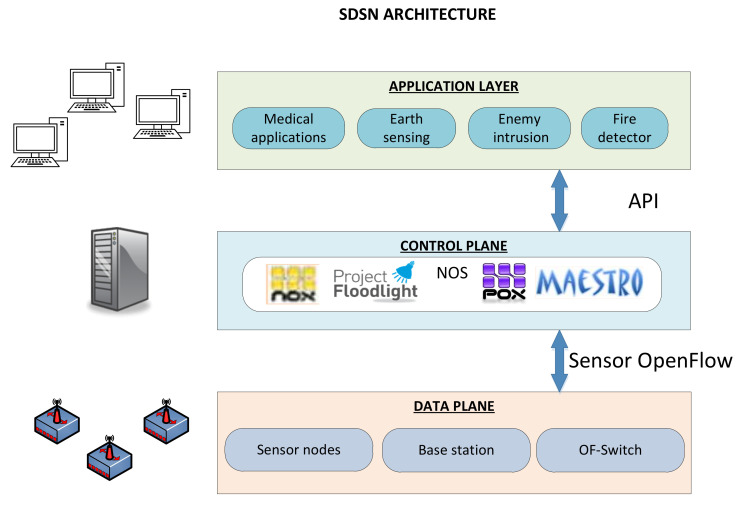
SDSN layers.

Logically, SDSNs are based on three software-definable layers in order to fulfill their functionality as Figure 5 depicts. The physical layer (data plane) is composed of sensor nodes and it additionally contains the Software Defined Radio (SDR) that is used to control the media access. The networking layer (control plane) is responsible for data transmission across the network and also the SDN concept (controller) is located within it. Finally, the application layer manages the role programs, sensing tasks and the operative system that controls sensor nodes.

### 4.2. Software Defined Sensor Networks Challenges

Therefore, SDSN get the best features of the union of WSNs and SDNs due to the design of Sensor OpenFlow [13]. One of the achieved advantages is the versatility since they are able to support several applications without installing any software (plug-and-play). Another benefit is the flexibility in terms of a quick change in the network behaviour due to the centralized control. In addition, the ease of management to control the network through an open Application Programming Interface (API) is a very important factor in these types of networks.

SDSNs have inspired several applications. Zeng et al. [62] propose the DASN project, which is the acronym for the Demand-Addressable Sensor Network. The purpose of this project is to build a sensor network that is able to perform sensing tasks based on user requirements. These demands are searched within sensor nodes and, if the requests are satisfied, such requests are mixed with more data in the network and are finally displayed to the user through a terminal.

SDSNs present some technical challenges [13] in that the research community tries to solve proposing several solutions. The first issue belongs to data flows (data plane), which are composed of packets and their addressing. In contrast to SDNs, WSNs do not use IP addresses, but employ addressing through attributes (e.g., sensor nodes over N atmospheres). The problem is solved with the inclusion of IP addressing (IPv4 [63] and IPv6 [64]) within WSNs. In the same way of the previous problem, the sensor OpenFlow channel [20] provides TCP/IP connectivity in the sending of data, and, as stated before, WSNs do not incorporate this addressing. An approach to give a solution to such a problem is using transport protocols over WSN, and, then, traffic will be delivered in its target. Moreover, since WSNs are sensitive to every change in the environment, the overhead of the network control traffic supposes a huge problem. Developing an algorithm that only sends the first packet when a table-miss is occurred, reduces the overhead of the network until the flow-mod message arrives. Finally, traffic generation is the most important issue in WSN since data packets that are sent from sensor nodes augment the traffic across the network. Adding a module that generates traffic in the network using synchronous, asynchronous and round-robin methods proposes a solution to control the amount of traffic in the network.

Mahmud et al. [65] propose an approach to use the OpenFlow technology within a WSN. Moreover, they expose four ideas to be developed in sensor networks in order to achieve an increment in reliability: firstly, it presents the concept of flow-sensor instead typical sensor that are being used in the present. Secondly, the use of the OpenFlow protocol in the communication between the flow-sensors and the controller. Thirdly, the traffic flow belonging to control messages (control plane) does not interfere in the data traffic (data plane). Finally, it intends to achieve a fool-proof routing in both traffic directions. Its main feature is the routing procedure to forward the data traffic. It assigns a value (cost) to all available routes in the network, and, after that, it saves each computed route in a tree structure. Once the routes’ tree has been calculated, the routing optimizer chooses the best route in terms of the number of hops that it is composed of. It will choose as the best route the one with a minimal number of hops. Nevertheless, if a problem occurs in a sensor node and it becomes inactive, the route optimizer will pick the second best route following the same choosing parameters.

Another challenge is the resource allocation and its management. In this way, Costanzo et al. [66] propose some ideas to manage network resources efficiently: data-aggregation only when is necessary, flexibility in routing paths and energy saving in sensor nodes when the radio is not working. To get these features, this architecture is layered including: an adaptation layer (packet formatting), virtualization layer (part of the network according to the terms of the topology) and a controller (working with flow table rules). Since there are obligatory data aggregations as the exchange messages between sensors and the controller when the network topology is being created, it is not clear if this architecture optimizes the energy use in sensor nodes.

Galluccio et al. propose SDN-WISE [67,68], an extension of Sensor OpenFlow [13]. It uses SDN in order to simplify policies to reconfigure WSN that are composed of distinct vendor sensor nodes. SDN-WISE is based on multiple controllers (local controllers) and an additional one (global controller) that works as a proxy between the data plane and them. Its main feature is that sensors only participate in local control tasks, removing the interaction with the global controller. Therefore, a packet can be treated in different ways since different flow rules are programmed within controllers.

Continuing along this line, Gante et al. [69] propose a solution to increment WSN management. The SDN controller is composed of a five-layer stack: the lower three layers are the physical layer, the MAC layer and finally the NOS layer. The controller is placed in the fourth layer, and the fifth is the Application layer where tasks such as routing, QoS and localization are managed. The authors affirm that this layered and centralized architecture achieves better performance in management tasks, and it also offers an energy savings in sensor nodes that belong to the release of the communication load.

As is stated in the previous section, the union between WSN and SDN architecture allows for multitasking in WSN. In this way, Zeng et al. [14,62] propose an architecture comprised of WSN and SDN allowing multitasking sharing the same network resources. Therefore, each sensor contains several programs running within them, which belongs to these applications based on user requirements. In order to support multitasking in an efficient way, it offers an optimization (in terms of load and energy) in all applications managed by a global controller with a scheduling process. This optimization process is defined as a minimum energy sensor activation problem taking into account restrictions on storage and coverage.

Table 2 gathers the SDN-based solutions for WSN and their features. The four right-side columns describe the features of each solution. A column named OpenFlow presents whether the proposed model is compatible with the OpenFlow protocol. Next, the following column is called Data Aggregation, which determines if the proposal aggregates data to the network. The management column shows if the solution manages network resources efficiently. Finally, the last column stated as Resource allocation indicates if the proposed solution is able to allocate network resources.

## 5. Wireless Cellular Networks

In this section, we overview the architecture of Wireless Cellular Networks and how SDNs can be added to this architecture to solve existing challenges. We describe these challenges under different categories and also the proposed solutions to them. Finally, this section concludes with a resume table where the proposed solutions described in this section are compared.

### 5.1. Wireless Cellular Networks Architecture

Wireless Cellular Networks (WCN) are composed of phones and smart devices that are connected to the Internet through base stations that are installed on cellular towers. At the same time, base stations are classified as Serving Gateway and Packet data network Gateway. These gateways work as control plane and data plane entities. On one hand, the control plane includes mobile connection establishment, routing, mobility management, QoS [15] and Quality of Experience (QoE). On the other hand, the data plane is comprised of access control and traffic monitoring. Nevertheless, the close union of both planes opens resource management and scalability challenges in WCN.

WCN architecture is comprised of several components; the main two components are RAN and Core Network (CN). RAN consists of close cellular towers that supply the access to user devices. Moreover, this technology offers functionality as resource allocation and essential management. For its part, CN provides connectivity between different RANs and also services such as connection establishment and maintenance, among others.

### 5.2. Wireless Cellular Network Challenges

Due to the current demand of WCN, data transferring has increased, resulting in a huge impact for user mobile experience [70] and resources. Then, this fact opens up a big challenge in terms of effective resource management. Several techniques are proposed to optimize resource allocation in order to achieve a better performance in WCN. In [71,72], strategies such as power control, cell splitting and sophisticated channel allocation are proposed. Depending on how these strategies are carried out, other challenges emerge: adequate QoS and QoE for voice and video traffic.

### 5.3. Software Defined Networks in Wireless Cellular Networks

Applying SDN in WCN removes the load of the Packet data network Gateway since decoupling data and control planes allows scalability and network manageability. At the same time, such facts reduce hardware components and also hardware cost. Consequently, having a centralized controller improves the performance and stability of the network achieving global resource allocation and interference management.

**Figure 6:**
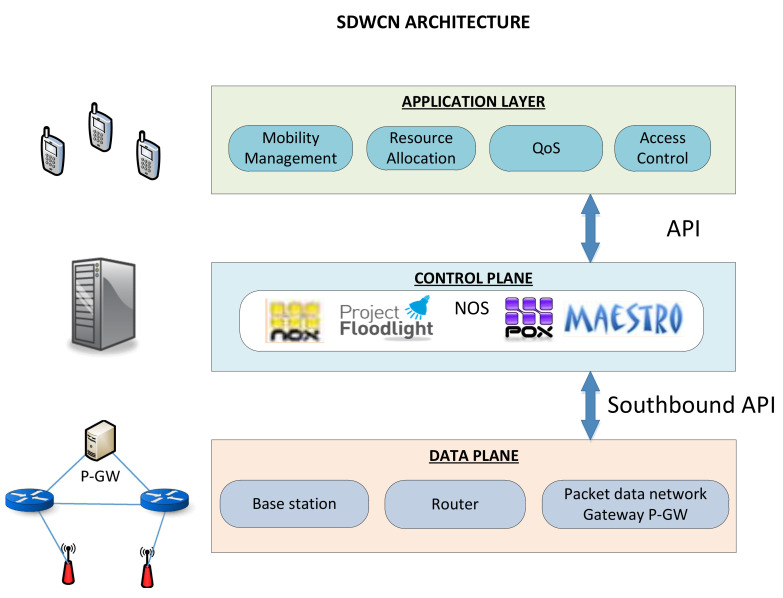
SDWCN layers.

The Software Defined Wireless Cellular Networks (SDWCN) design is comprised of the control plane (set of application modules that provide load balancing, QoS, mobility management, and connection establishment) and data plane (base stations and cellular towers), as is shown in Figure 6.

WCN physical deployment is based on sets of cells in order to provide communication services to network users. Therefore, phones must be within the access point coverage to be connected in such networks. Thus, WCNs use sets of cells to increase the network performance in terms of users’ traffic demands. Particularly, cells are divided into smaller ones that are administrated by access points. Due to this division, the interferences across cells eventually limit network capacity producing cells from overlapping. Hence, the paradigm of cell splitting (number of cells and their size) opens up difficult problems such as mobility management and load balancing in these networks [73].

Several works have been proposed using the SDN model in WCN to allocate resources in the best possible way [74,75,76]. We have divided these solutions into three different groups attending to the component of the WCN that is being improved. The first group belongs to works focused on RAN, the second group is targeted to CN, and, finally, solutions that use resource allocation through RAN and CN at the same time.

- Regarding resource allocation in RAN, works like SoftRAN [73] propose a centralized radio access control plane in order to abstract a group of base stations in a single virtual base station. It builds a three-dimensional grid of resources based on abstraction of space, time and frequency dimensions. Nevertheless, this design takes into account the network delay even when the resource allocation is performed quickly. Hence, the logical centralized control is responsible for global network control aspects, while the data plane devices are handled by a tasks distribution process. Moreover, SoftRAN is able to improve the handover process and resource allocation based on interferences and also assigns power to resources that are placed at a base station in a dense WCN. Chen et al. propose SoftMobile [77], an approach that provides an alternative tasks’ distribution to solve three sub-problems: how can state information be distributed, how can the cells be coherently configured, and, finally, how can live inter-cell operations be handled. Each one of these problems are managed by three separated controllers.
- There exists a need of resource allocation at the cellular CN where RAN connects devices to the Internet. Traditional cellular networks centralize this resource allocation at the base station. Packet data network Gateways are responsible for checking the resource availability to satisfy a resource demand with priority that belongs to a user or an application or taking the correspondent actions (denying request or releasing the resource). This model raises scalability problems requiring expensive Packet data network Gateways to satisfy this challenge. In this way, SoftCell [78] proposes a solution to remove the complexity from Packet data network Gateways in order to access switches placed at base stations. Nevertheless, this solution presents important bandwidth requirements that are translated as congestion and also a limitation of network scalability. To meet this matter, SoftCell presents an algorithm that aggregates multidimensional packets in order to decrease the forwarding table size in data plane devices.
- Regarding solutions that use programmable RAN and CN, the resource allocation challenge is present both in RAN and in the CN. SoftAir [79] is a wireless network architecture for 5G based on the SDN model. Its control plane is comprised of two components: network management tools and customized applications that provide services to network operators. For its part, the data plane is based on CN switches and base stations that use Software Defined Radio Access Networks. The programmable CN offers efficient network virtualization and traffic classification. Concretely, it uses a three-way virtualization of the network that guarantees effective and non-conflicting allocation of network resources among network operators. Traffic classification is divided into local classification (performed by base stations) and global classification (performed by the controller). Another challenge is the mobility management that SoftAir performs in two steps: location management and QoS handoff-rerouting. Handoff-rerouting is performed exploiting the global network view that the centralized controller gets. The union between optimized placement and the mobility pattern of users establishes an optimal control traffic path from data plane devices through the controller.
- A variation of Softair architecture is MyNet [80], a hierarchical deployment of controllers for 5G [81] networks that are responsible for each part of the network in order to reduce the control traffic flow. Yazici et al. [82] also propose a hierarchical architecture titled CMaaS that manifests a four-layer controller hierarchy. The bottom layer, called a UE controller, is responsible for managing the available radio access technology selection for a user by local network status. The next layer is called a BS controller that manages the time-sensitive radio resource management and scheduling with a local network view. Continuing with the layered architecture, the RAN controller controls the set of base stations obtaining a regional view. The highest layer, called a Network controller, offers a global network view and manages services such as QoS, mobility management and routing. Control decisions are distributed from lower layers to upper layers. Parallel, upper layers get network information from controllers to gather a global state view and make control decisions.

Table 3 gathers the SDN-based solutions for WCN and their features. The four right-side columns describe the properties of each solution. A column named OpenFlow presents if the proposed model is compatible with OpenFlow protocol. Next, the following column is called Scalability, which determines if the proposal is able to be scalable in terms of network size. A Data Plane column shows which data plane component is being improved in the proposal (RAN, CN or both). Finally, the last column stated as Resource allocation indicates if the proposed solution is able to allocate network resources.

## 6. Conclusions and Future Works

### 6.1. Conclusions

We have shown in this paper a review and a taxonomy of existing Software Defined Networking (SDN) technology. SDN is considered to be one of the most important advances in network technologies due to its revolutionary architecture and the network management that it offers. It also plays an important role in other kinds of technologies, improving its performance, reliability and scalability. Different technologies and their main challenges have been discussed in this paper. They differ in terms of organization but look for the same features: resource allocation and efficient management of the network. In conclusion, decoupling both planes (control and data) provides advantages and solutions for each technology presented in this paper. Next, we resume the primary conclusions for each technology and the solutions to their challenges including the SDN model.

SDN has removed the rigidity in the traditional networks decoupling control and data planes. Moreover, SDN has managed the constant changing of functions and improved a unified view within the network. Therefore, SDN provides scalability, flexibility, and centralized control to achieve the best performance in the network. Virtualization is identified as an integrated component of programmable networks where different networks can share the same physical underlying infrastructure. Then, there is a need to achieve an efficient virtualization strategy since it is an open challenge.

WSNs have been developed due to the need of executing specific applications. Moreover, WSNs have attracted the research community interest due to its versatility to execute several applications using the same hardware. Environmental monitoring, health monitoring and industrial monitoring are the main applications of WSNs. The main field of WSN applicability is the environmental monitoring among others as earth sensing monitoring, air pollution, fire detection systems, natural disasters detection systems [51], and volcano eruption systems [52]. Therefore, WSNs are very used to monitoring environments due to its versatility and mobility to be deployed everywhere. Regarding the health care field, medical applications [49] are able to detect diseases in an early stage in order to perform a treatment to the person that is being monitored. Security in WSN [49] is also important to send data across the network in a secure way.

The union of WSN and SDN into SDSN has supposed important progress in the field of environmental monitoring topologies. We have described three types of SDN-based solutions in terms of their features: multi-application, energy optimization and task distribution. Regarding multi-application solutions [14], Sensor OpenFlow [13] is a SDN-based solution sensor network that provides multi-application environment on a physical network and also a compatible protocol to support multi-vendor data plane resources. Regarding energy optimization, SDN-WISE [67] is an extension of Sensor OpenFlow that looks for an efficient energy use through cycle of works and data aggregation. The last group belongs to task distribution solutions. We have included in this group a proposal called Smart [69] that offers an optimized energy use through task distribution between data and control planes.

Regarding Wireless Cellular Networks, we have described its architecture and the main problems that present this kind of network. Moreover, SDN solutions have been proposed to solve the challenges as resource allocation and scalability. These solutions have been divided into two groups. The first group is focused on RAN and looks for the performance enhancement and scalability over an efficient resource allocation. One solution is SoftRAN [73] that is focused on RAN and its programmability improving the resource management, mobility and load balancing. These improvements are achieved by removing some control traffic sent from the centralized controller to data plane resources. This group also includes a SoftMobile [77] solution that provides task distribution through a layered controller design. Lower layers are responsible for managing local changes while upper layers perform control actions. The second group is focused on the CN and an efficient resource allocation. SoftCell [78] removes the complexity from Packet data network Gateways in order to access data plane resources (switches). The third and last group is comprised of solutions that use RAN and CN at the same time. SoftAir [79], MyNet [80] and CMaaS [82] are hierarchical architectures based on SDN for 5G networks that provide less control traffic and they also provide services as QoS and QoE.

### 6.2. Future Works

Taking into account that each application needs different requirements, the development of new designs, new protocols, new services, among others, in order to fulfill such requirements is needed. Services for controlling and management include data aggregation, security, synchronization and cross-layer optimization. In a huge WSN, time synchronization can be a serious problem since the data that is not updated at a specific point of time can produce event collision and energy wastage, among others. Therefore, there is a need to maintain time synchronization across the network. Furthermore, transferring huge amounts of data generated over the network to the base station is quite expensive. We have described some solutions that look for reducing the amount of data to be transferred as well as an efficient use of sensor nodes. In these terms, future works of data optimization and energy saving will be crucial to obtaining a balanced WSN.

Secure WSN protocols have to monitor, detect and respond to attackers without interrupting the application or service that are offering at the same time. Cross-layer secure monitoring in a WSN is an important challenge that has to be fixed.

The union of WSN and SDN into SDSN has supposed an important progress in the field of environmental monitoring topologies. It allows for controlling one or multiple applications that are executed in the topology without much effort due to the centralized controller that SDN technology provides in its architecture. Definitely, next-generation sensor networks will be managed by a controller due to the efficient control and management solutions that they provide, and, at the same time, their replacement procedure is quite simple.

## Figures and Tables

**Table 1:** NOS grouped by the language that are programmed.

Language	Controller Name
C/C++	NOX, Trema, MUL
Haskell	Nettle, McNettle, NetCore
Java	Maestro, Floodlight, Beacon, ONOS, OpenDayLight
OCaml	Mirage, Frenetic
Python	POX, Pyretic, RYU

**Table 2:** Differences between Software Defined Sensor Networks (SDSNs).

Reference	Proposal	OpenFlow	Data Aggregation	Management	Resource Allocation
[13]	Sensor OpenFlow	✓	✓	✓	X
[62]	Multitasking SDN	X	X	✓	✓
[65]	Explotation OpenFlow	✓	✓	✓	X
[66]	SDWN	✓	X	✓	✓
[67]	SDN-WISE	✓	✓	✓	✓
[69]	Smart WSN	X	X	✓	X

**Table 3:** Differences between Software Defined Sensor Networks.

Reference	Proposal	OpenFlow	Scalability	Data Plane	Resource Allocation
[73]	SoftRAN	X	X	RAN	✓
[77]	SoftMobile	X	X	RAN	✓
[78]	SoftCell	X	✓	CN	X
[79]	SoftAir	✓	X	RAN and CN	✓
[80]	MyNet	X	X	RAN and CN	✓
[82]	CMaaS	✓	X	RAN	✓

## Abbreviations

The following abbreviations are used in this manuscript:

API	Application Programming Interface
CN	Core Network
CSMA/CA	Carrier Sense Multiple Access/Collision Avoidance
DASN	Demand-Addressable Sensor Network
DoS	Denial of Service
DDoS	Distributed Denial of Service
GPP	General Purpose Processor
IP	Internet Protocol
IoT	Internet of Things
MAC	Medium Access Control
MEC	Multi-Access Edge Computing
NFP	Network Flow Processor
NFV	Network Function Virtualization
NETCONF	Network Configuration Protocol
NOS	Network Operative System
ONF	Open Networking Foundation
ONOS	Open Network Operating System
OS	Operative System
QoE	Quality of Experience
QoS	Quality of Service
RAN	Radio Access Network
SCS	Sensor Control Server
SDN	Software Defined Network
SDR	Software Defined Radio
SDSN	Software Defined Sensor Network
SDWCN	Software Defined Wireless Cellular Network
SNMP	Simple Network Management Protocol
TLS	Transport Layer Security
VLAN	Virtual Local Area Networks
WCN	Wireless Cellular Network
WSN	Wireless Sensor Network

## References

[1] Schwartz B., Jackson A.W., Strayer W.T., Zhou W., Rockwell R.D., Partridge C. Smart Packets for Active Networks. Proceedings of the 1999 IEEE Second Conference on Open Architectures and Network Programming Proceedings.

[2] Schwartz B., Jackson A.W., Strayer W.T., Zhou W., Rockwell R.D., Partridge C. (2000). Smart Packets: Applying Active Networks to Network Management. ACM Trans. Comput. Syst..

[3] Sherwood R., Gibb G., Yap K.K., Appenzeller G., Casado M., McKeown N., Parulkar G. (2009). Flowvisor: A Network Virtualization Layer. OpenFlow Switch Consort..

[4] Barona López L.I., Valdivieso Caraguay A.L., García Villalba L.J., López D. (2015). Trends on Virtualisation with Software Defined Networking and Network Function Virtualisation. IET Netw..

[5] Ofelia Consortium. http://www.fp7-ofelia.eu/.

[6] Open Networking Foundation. http://www.opennetworking.org.

[7] Bonomi F., Milito R., Zhu J., Addepalli S. Fog computing and its role in the internet of things. Proceedings of the First Edition of the MCC Workshop on Mobile Cloud Computing.

[8] Yi S., Li C., Li Q. A survey of Fog Computing: Concepts, Applications and Issues. Proceedings of the Workshop on Mobile Big Data.

[9] Gupta H., Nath S.B., Chakraborty S., Ghosh S.K. (2016). SDFog: A Software Defined Computing Architecture for QoS Aware Service Orchestration over Edge Devices. arXiv.

[10] Taleb T., Samdanis K., Mada B., Flinck H., Dutta S., Sabella D. (2017). On multi-access edge computing: A survey of the emerging 5G network edge cloud architecture and orchestration. IEEE Commun. Surv. Tutor..

[11] Akyildiz I.F., Su W., Sankarasubramaniam Y., Cayirci E. (2002). Wireless sensor networks: A survey. Comput. Netw..

[12] Yick J., Mukherjee B., Ghosal D. (2008). Wireless sensor network survey. Comput. Netw..

[13] Luo T., Tan H.P., Quek T.Q. (2012). Sensor OpenFlow: Enabling software-defined wireless sensor networks. IEEE Commun. Lett..

[14] Zeng D., Li P., Guo S., Miyazaki T., Hu J., Xiang Y. (2105). Energy minimization in multi-task software-defined sensor networks. IEEE Trans. Comput..

[15] Li L.E., Mao Z.M., Rexford J. Toward software-defined cellular networks. Proceedings of the 2012 European Workshop on Software Defined Networking (EWSDN).

[16] IEEE Standard for Wireless LAN Medium Access Control (MAC) and Physical Layer (PHY) Specifications. http://standards.ieee.org/findstds/standard/802.11-1997.html.

[17] IEEE Standard for Local and Metropolitan Area Networks—Part 16: Air Interface for Fixed Broadband Wireless Access Systems. http://standards.ieee.org/findstds/standard/802.16-2001.html.

[18] Valdivieso Caraguay A.L., Benito Peral A., Barona López L.I., García Villalba L.J. (2014). SDN: Evolution and Opportunities in the Development IoT Applications. Int. J. Distrib. Sens. Netw..

[19] McKeown N., Anderson T., Balakrishnan H., Parulkar G., Peterson L., Rexford J., Shenker S., Turner J. (2008). OpenFlow: Enabling Innovation in Campus Networks. ACM SIGCOMM Comput. Commun. Rev..

[20] OpenFlow Switch Specification v1.1.0. http://archive.openflow.org/documents/openflow-spec-v1.1.0.pdf.

[21] Chowdhury N., Boutaba R. (2010). A survey of network virtualization. Comput. Netw..

[22] Sherwood R., Gibb G., Yap K.K., Appenzeller G., Casado M., McKeown N., Parulkar G.M. Can the production network be the testbed?. Proceedings of the USENIX Symposium on Operating Systems Design and Implementation (OSDI).

[23] Jin X., Gossels J., Rexford J., Walker D. CoVisor: A compositional hypervisor for software-defined networks. Proceedings of the 12th USENIX Symposium on Networked Systems Design and Implementation (NSDI 15).

[24] Han B., Gopalakrishnan V., Ji L., Lee S. (2015). Network function virtualization: Challenges and opportunities for innovations. IEEE Commun. Mag..

[25] Mijumbi R., Serrat J., Gorricho J.L., Bouten N., De Turck F., Boutaba R. (2016). Network function virtualization: State-of-the-art and research challenges. IEEE Commun. Surv. Tutor..

[26] Reitblatt M., Foster N., Rexford J., Schlesinger C., Walker D. Abstractions for Network Update. Proceedings of the ACM SIGCOMM 2012 Conference on Applications, Technologies, Architectures, and Protocols for Computer Communication.

[27] Sezer S., Scott-Hayward S., Chouhan P.K., Fraser B., Lake D., Finnegan J., Rao N. (2013). Are We Ready for SDN? Implementation Challenges for Software-defined Networks. IEEE Commun. Mag..

[28] Kim H., Feamster N. (2013). Improving Network Management with Software Defined Networking. IEEE Commun. Mag..

[29] Gude N., Koponen T., Pettit J., Pfaff B., Casado M., McKeown N., Shenker S. (2008). NOX: Towards an Operating System for Networks. ACM SIGCOMM Comput. Commun. Rev..

[30] Cai Z., Cox A.L., Eugene T.S. (2010). Maestro: A System for Scalable OpenFlow Control.

[31] Beacon: A Java-Based OpenFlow Control Platform. http://www.beaconcontroller.net.

[32] Project Floodlight: Open Source Software for Building Software-Defined Networks. http://www.projectfloodlight.org.

[33] Project ONOS: Open Network Operating System for Building Software-Defined Networks. https://onosproject.org.

[34] OpenDaylight (ODL) Open Source SDN Platform. https://www.opendaylight.org.

[35] Foster N., Harrison R., Freedman M.J., Monsanto C., Rexford J., Story A., Walker D. (2011). Frenetic: A Network Programming Language. ACM Sigplan Not..

[36] Foster N., Guha A., Reitblatt M., Story A., Freedman M.J., Katta N.P., Walker D. (2013). Languages for Software-defined Networks. IEEE Commun. Mag..

[37] Voellmy A., Kim H., Feamster N. Procera: A Language for High-level Reactive Network Control. Proceedings of the First Workshop on Hot Topics in Software Defined Networks.

[38] Monsanto C., Foster N., Harrison R., Walker D. A Compiler and Run-time System for Network Programming Languages. Proceedings of the 39th Annual ACM SIGPLAN-SIGACT Symposium on Principles of Programming Languages.

[39] Voellmy A., Wang J. Scalable Software Defined Network Controllers. Proceedings of the ACM SIGCOMM 2012 Conference on Applications, Technologies, Architectures, and Protocols for Computer Communication.

[40] Ozdag R. Intel Ethernet Switch FM6000 Series—Software Defined Networking. http://www.intel.co.uk/content/dam/www/public/us/en/documents/white-papers/ethernet-switch-fm6000-sdn-paper.pdf.

[41] Netronome NFP6XXX Flow Processor. http://netronome.com/pages/flow-processors/.

[42] Tootoonchian A., Ganjali Y. HyperFlow: A Distributed Control Plane for OpenFlow. Proceedings of the 2010 Internet Network Management Conference on Research on Enterprise Networking.

[43] Scott-Hayward S., O’Callaghan G., Sezer S. Sdn security: A survey. Proceedings of the Future Networks and Services (SDN4FNS).

[44] Duan Q., Ansari N., Toy M. (2016). Software-Defined Network Virtualization—An Architectural Framework for Integrating SDN and NFV for Service Provisioning in Future Networks. IEEE Netw. Mag..

[45] Zanjireh M.M., Larijani H. A Survey on Centralised and Distributed Clustering Routing Algorithms for WSNs. Proceedings of the 2015 IEEE 81st Vehicular Technology Conference (VTC Spring).

[46] OPNET Network Simulator. http://es.riverbed.com/products/performance-management-control/opnet.html.

[47] NetSim—Network Simulator. http://tetcos.com.

[48] NS2—Network Simulator. http://www.isi.edu/nsnam/ns/.

[49] Gao T., Greenspan D., Welsh M., Juang R.R., Alm A. Vital signs monitoring and patient tracking over a wireless network. Proceedings of the 2005 IEEE Engineering in Medicine and Biology 27th Annual Conference.

[50] Lorincz K., Malan D., Fulford-Jones T.R.F., Nawoj A., Clavel A., Shnayder V., Mainland G., Welsh M., Moulton S. (2004). Sensor networks for emergency response: Challenges and opportunities, Pervasive Computing for First Response (Special Issue). IEEE Pervasive Comput..

[51] Castillo-Effen M., Quintela D.H., Jordan R., Westhoff W., Moreno W. Wireless sensor networks for flash-flood alerting. Proceedings of the Fifth IEEE International Caracas Conference on Devices, Circuits and Systems.

[52] Werner-Allen G., Lorincz K., Ruiz M., Marcillo O., Johnson J.M., Lees J., Welsh M. (2006). Deploying a wireless sensor network on an active volcano. IEEE Internet Comput..

[53] García Villalba L.J., Sandoval Orozco A.L., Triviño Cabrera A., Barenco Abbas C.J. (2009). Routing protocols in wireless sensor networks. Sensors.

[54] Chong C.Y., Kumar S.P. (2003). Sensor networks: Evolution, opportunities, and challenges. Proc. IEEE.

[55] Baronti P., Pillai P., Chook V.W., Chessa S., Gotta A., Hu Y.F. (2007). Wireless sensor networks: A survey on the state of the art and the 802.15.4 and ZigBee standards. Comput. Commun..

[56] Institute of Electrical and Electronics Engineers, Inc. (2003). IEEE Standard for Information Technology—Wireless Medium Access Control (MAC) and Physical Layer (PHY) Specifications for Low Rate Wireless Personal Area Networks (LR-WPANs).

[57] Anastasi G., Conti M., Di Francesco M. (2011). A comprehensive analysis of the MAC unreliability problem in IEEE 802.15.4 wireless sensor networks. IEEE Trans. Ind. Inform..

[58] Perrig A., Stankovic J., Wagner D. (2004). Security in wireless sensor networks. Commun. ACM.

[59] Wood A., Stankovic J. (2002). Denial of service in sensor networks. IEEE Computer..

[60] Karlof C., Wagner D. Secure routing in wireless sensor networks: Attacks and countermeasures. Proceedings of the 1st IEEE International Workshop on Sensor Network Protocols and Applications.

[61] Ye W., Heidemann J., Estrin D. An energy-efficient MAC protocol for wireless sensor networks. Proceedings of the INFOCOM 2002, Twenty-First Annual Joint Conference of the IEEE Computer and Communications Societies.

[62] Zeng D., Miyazaki T., Guo S., Tsukahara T., Kitamichi J., Hayashi T. Evolution of software-defined sensor networks. Evolution of software-defined sensor networks. Proceedings of the Mobile Ad-hoc and Sensor Networks (MSN), 2013 IEEE Ninth International Conference.

[63] Internet Protocol. RFC 791. https://tools.ietf.org/html/rfc791.

[64] Internet Protocol, Version 6 (IPv6) Specification. RFC 2460. https://tools.ietf.org/html/rfc2460.

[65] Mahmud A., Rahmani R. Exploitation of OpenFlow in wireless sensor networks. Proceedings of the 2011 International Conference on Computer Science and Network Technology (ICCSNT).

[66] Costanzo S., Galluccio L., Morabito G., Palazzo S. Software defined wireless networks: Unbridling SDN. Proceedings of the 2012 European Workshop on Software Defined Networking.

[67] Galluccio L., Milardo S., Morabito G., Palazzo S. SDN-WISE: Design, prototyping and experimentation of a stateful SDN solution for WIreless SEnsor networks. Proceedings of the 2015 IEEE Conference on Computer Communications (INFOCOM).

[68] Galluccio L., Milardo S., Morabito G., Palazzo S. Reprogramming wireless sensor networks by using SDN-WISE: A hands-on demo. Proceedings of the IEEE Conference on Computer Communications Workshops (INFOCOM WKSHPS).

[69] Gante A.D., Aslan M., Matrawy A. Smart wireless sensor network management based on software-defined networking. Proceedings of the 27th Biennial Symposium on Communications (QBSC).

[70] Cisco (2015). Cisco Visual Networking Index: Global Mobile Data Traffic Forecast Update 2014–2019.

[71] Agbinya J.I., Aguayo-Torres M.C., Klempous R. (2013). 4G Wireless Communication Networks: Design Planning and Applications.

[72] Damnjanovic A., Montojo J., Wei Y., Ji T., Luo T., Vajapeyam M., Yoo T., Song O., Malladi D. (2011). A survey on 3GPP heterogeneous networks. IEEE Wirel. Commun..

[73] Gudipati A., Perry D., Li L., Katti S. SoftRAN: Software defined radio access network. Proceedings of the Second ACM SIGCOMM Workshop on Hot Topics in Software Defined Networking.

[74] Kobo H.I., Abu-Mahfouz A.M., Hancke G.P. (2017). A Survey on Software-Defined Wireless Sensor Networks: Challenges and Design Requirements. IEEE Access.

[75] Modieginyane K.M., Letswamotse B.B., Malekian R.M., Abu-Mahfouz A.M. (2017). Software defined wireless sensor networks application opportunities for efficient network management: A survey. Comput. Electr. Eng..

[76] Ndiaye M., Hancke G.P., Abu-Mahfouz A.M. (2017). Software Defined Networking for Improved Wireless Sensor Network Management: A Survey. Sensors.

[77] Chen T., Zhang H., Chen X., Tirkkonen O. (2014). SoftMobile: Control evolution for future heterogeneous mobile networks. IEEE Wirel. Commun..

[78] Jin X., Li L.E., Vanbever L., Rexford J. Softcell: Scalable and flexible cellular core network architecture. Proceedings of the Ninth ACM Conference on Emerging Networking Experiments and Technologies.

[79] Akyildiz I.F., Wang P., Lin S.C. (2015). SoftAir: A software defined networking architecture for 5G wireless systems. Comput. Netw..

[80] Zhang H., Vrzic S., Senarath G., Dào N.D., Farmanbar H., Rao J., Peng C., Zhuang H. (2015). 5G wireless network: MyNET and SONAC. IEEE Netw..

[81] Santos J.P., Alheiro R., Andrade L., Valdivieso Caraguay Á.L., Barona López L.I., Sotelo Monge M.A., García Villalba L.J., Jiang W., Schotten H., Alcaraz Calero J.M. (2016). SELFNET Framework self-healing capabilities for 5G mobile networks. Trans. Emerg. Telecommun. Technol..

[82] Yazici V., Kozat U.C., Sunay M.O. (2014). A new control plane for 5G network architecture with a case study on unified handoff, mobility, and routing management. IEEE Commun. Mag..

